# Entecavir-Induced Lichenoid Drug Eruption: A Case Report

**DOI:** 10.7759/cureus.108696

**Published:** 2026-05-12

**Authors:** Sushantika Sushantika, Piyush Yadav, Jyoti Sethi, Soumya Nanda, Kartik Saini

**Affiliations:** 1 Dermatology, All India Institute of Medical Sciences, Gorakhpur, IND; 2 Dermatology, Rajasthan University of Health Sciences (RUHS) College of Medical Sciences, Jaipur, IND; 3 Dermatology, All India Institute of Medical Sciences, Rishikesh, IND

**Keywords:** antiviral drug, cutaneous adverse drug reaction, entecavir, hepatitis b infection, lichenoid drug eruption

## Abstract

Entecavir is a highly potent antiviral medication used in the treatment of chronic hepatitis B infection and has demonstrated an excellent safety profile in clinical practice. Cutaneous adverse reactions to entecavir are exceedingly rare, with only seven cases reported in the literature to our knowledge. Here, we report the case of an adult female receiving entecavir therapy for chronic hepatitis B who developed a lichenoid drug eruption secondary to the medication. The rash resolved promptly following drug discontinuation and supportive treatment. Various cutaneous morphological patterns associated with entecavir have been reported in the literature, including immediate hypersensitivity reactions, maculopapular rash, granulomatous eruptions, erythematous plaques, bullous fixed drug eruption, and lichenoid erythematous patches. Clinicians should remain aware of the potential for cutaneous adverse drug reactions associated with entecavir and their varied morphological presentations to facilitate prompt recognition and appropriate management.

## Introduction

Entecavir is a highly potent antiviral agent approved by the US Food and Drug Administration for the treatment of chronic hepatitis B infection in adults and children older than two years of age [1]. It is a selective inhibitor of DNA polymerase with high antiviral efficacy. Entecavir is recommended at a dose of 0.5 mg orally once daily in adults (and children older than 16 years) with typical chronic hepatitis B, and 1.0 mg once daily for patients with lamivudine-resistant HBV infection. Common adverse effects of entecavir include elevated alanine aminotransferase levels, lactic acidosis, upper respiratory tract infections, headache, abdominal pain, fever, fatigue, and diarrhea [2]. However, cutaneous adverse effects of entecavir are rare, with only seven cases reported to date. Herein, we report a case of lichenoid drug eruption in a patient receiving entecavir therapy for chronic hepatitis B.

## Case presentation

**Figure 1:**
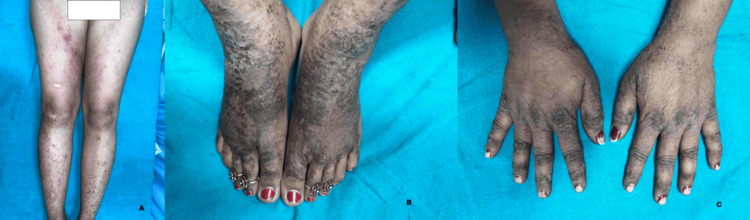
Multiple lichenoid papules and plaques with a violaceous hue and surrounding erythema present over the bilateral dorsum feet, shin, inner thigh, buttocks, dorsum hands, and forearm.

A 39-year-old female patient presented with complaints of a rash for the past month. She was a known case of mixed autoimmune hemolytic anemia and had been diagnosed with chronic hepatitis B infection two months earlier. The patient was receiving oral prednisolone 25 mg for autoimmune hemolytic anemia and had been started on oral entecavir 0.5 mg two months earlier for the treatment of chronic hepatitis B. Approximately one month after initiation of therapy, the patient developed multiple erythematous pruritic lesions involving the bilateral upper and lower limbs, buttocks, and abdomen. The lesions gradually increased in size, became thickened, and darkened over time. There were no other systemic complaints. On dermatological examination, multiple papules and plaques with a violaceous hue and surrounding erythema were present over the bilateral dorsum of the feet, shins, inner thighs, buttocks, dorsum of the hands, and forearms, with sparing of the face, mucosa, nails, palms, and soles (Figure 1). The lesions over the dorsum of the hands and feet, and around the ankles, showed lichenification. No associated lymphadenopathy was noted. There were no other known comorbidities, and no history of any other drug intake or topical application prior to the onset of the rash.

**Figure 2:**
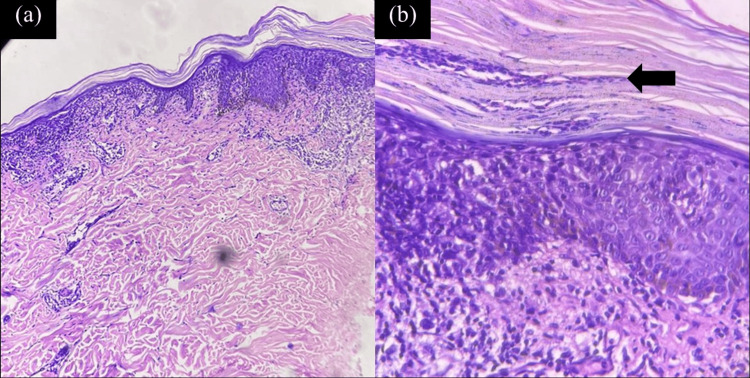
Histopathological examination (H&E stain) showing spongiosis with lichenoid infiltrate at the dermoepidermal junction, with mild periadnexal inflammatory infiltrates.

Routine blood work showed mildly elevated aspartate aminotransferase, alanine aminotransferase, and alkaline phosphatase levels. The patient underwent a skin biopsy from a papular lesion over the shin. Histopathological examination (H&E stain) revealed spongiosis with a lichenoid infiltrate at the dermoepidermal junction, along with mild periadnexal inflammatory infiltrates (Figure 2). Based on the clinical history, physical examination, laboratory findings, and histopathological features, a diagnosis of lichenoid drug eruption secondary to entecavir was made.

**Figure 3:**
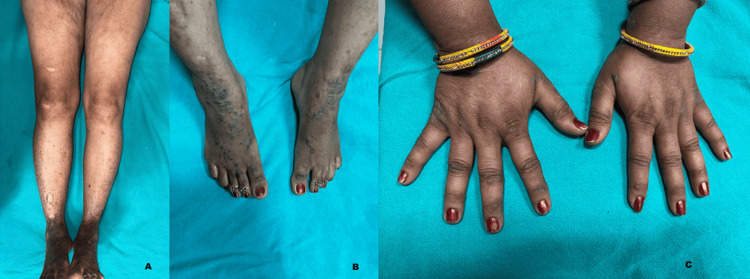
The lichenoid lesions improved remarkably after two months of cessation of entecavir and initiation of corticosteroids.

Entecavir was discontinued immediately, and the patient was switched to tenofovir for chronic hepatitis B management. For dermatological symptoms, the patient was started on a short course of oral corticosteroids at a dose of prednisolone 30 mg/day, given for a total duration of two months in a tapering regimen, along with topical clobetasol propionate 0.05% and emollient applications. The lesions showed marked improvement after two months following discontinuation of entecavir and initiation of corticosteroid therapy (Figure 3). The patient was continued on an alternative antiviral agent, with no further adverse effects reported.

## Discussion

Entecavir is a potent antiviral agent with a well-established safety profile, making it an important therapeutic option for the treatment of chronic hepatitis B worldwide. Despite its favorable safety profile, several adverse effects have been reported, including elevated alanine aminotransferase levels, lactic acidosis, upper respiratory tract infections, headache, abdominal pain, fever, fatigue, and diarrhea [3]. However, cutaneous adverse reactions associated with entecavir are very rare. Reported manifestations include immediate allergic skin reactions [4], maculopapular rash [5,6], granulomatous eruption [7], erythematous plaques [8], bullous fixed drug eruption [9], and lichenoid erythematous patches [10].

The etiopathogenesis of cutaneous eruptions secondary to entecavir remains unclear. A reduction in regulatory T cells or alterations in T-helper cell activity during entecavir treatment have been proposed as a possible mechanism [10]. Yamada et al. postulated that a decrease in regulatory T cells during the early phase of entecavir therapy, along with an increase in IL-17-producing T-helper cells, may contribute to the development of these cutaneous reactions [5]. Cytokine release assay profiles were also studied by Jeong et al. [6]. The authors observed increased IL-4 levels at 18 and 24 hours after entecavir treatment, while levels of other cytokines (IL-2, IL-6, IL-8, and TNF-α) were also elevated, although the differences from controls were not statistically significant.

Another interesting observation highlighted in previous reports is that all eight cases reported to date, including our case, originated from Asian countries. This raises the possibility of a genetic predisposition to entecavir-induced drug eruptions. However, the association between specific HLA alleles and entecavir-induced cutaneous reactions has yet to be established.

To our knowledge, only seven cases had been reported previously; therefore, our case represents the eighth reported case (Appendix 1). Our patient presented with multiple erythematous patches and raised plaques over the extremities and trunk, which differed from the presentations described in previous case reports. The onset of cutaneous eruptions occurred one month after initiation of entecavir therapy, which is consistent with previous reports describing onset intervals ranging from two days to six months [8]. The absence of any recent exposure to other drugs, along with the clinical and histopathological findings and the marked resolution of lesions following drug discontinuation, supported the diagnosis of entecavir-induced lichenoid drug eruption. Treatment consisted of immediate discontinuation of entecavir along with oral and topical corticosteroids. Oral corticosteroid therapy was considered appropriate in our patient because of the extensive cutaneous involvement, with satisfactory clinical improvement observed thereafter.

## Conclusions

In conclusion, we report a case of lichenoid drug eruption secondary to entecavir in a patient with chronic hepatitis B. The spectrum of cutaneous adverse reactions associated with entecavir continues to expand, with various morphologies already being described in the literature. Clinicians should be aware that cutaneous eruptions can significantly affect patient compliance with treatment regimens, potentially leading to worsening of the underlying disease. Therefore, awareness of the spectrum of entecavir-induced cutaneous eruptions and their prompt recognition and management is essential for clinicians.

## Appendices

Appendix 1 

**Table 1:** Summary of cases of drug eruption secondary to entecavir

S. no.	Author (year)	Age (in years)	Sex	Clinical manifestations	Sites of lesions	Time interval	Confirmed by histopathology
1	Sugiura et al. (2009) [4]	30	Male	Immediate allergy	Buttock	2 days	Not done
2	Yamada et al. (2011) [5]	62	Male	Maculopapular rash	Trunk and extremities	7 days	Not done
3	Yoon et al. (2013) [7]	65	Female	Granulomatous eruption	Forehead and face	2 months	Yes
4	Bellini et al. (2014) [8]	65	Male	Erythematous plaque	Upper limbs	6 months	Yes
5	Jeong et al. (2016) [6]	45	Male	Maculopapular rash	Back and extremities	1 month	Yes
6	Temiz et al. (2019) [9]	50	Female	Bullous eruption	Lower limb	7 days	Yes
7	Cheong et al. (2020) [10]	55	Male	Lichenoid erythematous patch	Trunk and extremities	6 weeks	Yes
8	Our case (2025)	39	Female	Lichenoid eruption	Trunk and extremities	1 month	Yes
